# Hypertensive Crisis: The Other Side of Polyarteritis Nodosa

**DOI:** 10.7759/cureus.6775

**Published:** 2020-01-26

**Authors:** Fahad Alsohime, Talal AlHoshan, Faisal Almojel, Nader A Rezeni, Mohamad-Hani Temsah

**Affiliations:** 1 Pediatric Intensive Care Unit, King Khalid University Hospital, Riyadh, SAU; 2 Pediatric Intensive Care Unit, King Khalid Unversity Hospital, Riyadh, SAU

**Keywords:** polyarteritis nodosa, hypertensive crisis, picu, pediatrics

## Abstract

Hypertensive crisis is a deadly complication that should be avoided at all costs, let alone when it is associated with a rare disease, such as polyarteritis nodosa. We present a case of a child who initially came to our hospital with a prolonged high-grade fever responding to antipyretics. Upon follow up, the patient went into a hypertensive crisis, which led to the admission to the Pediatric Intensive Care Unit (PICU). The hypertension was difficult to control until the culprit was revealed. Polyarteritis nodosa was then diagnosed by angiography, which revealed tight stenosis of the right renal artery. Prompt treatment was initiated, and the patient's blood pressure normalized. Our case highlights the importance of detecting such diseases, since it may be easily missed as it usually presents with nonspecific symptoms. Hypertension in children shouldn't be taken lightly and should be investigated promptly. We hope our case report sheds enough light on this issue for other clinicians and researches to identify and prevent later on.

## Introduction

Polyarteritis nodosa (PAN) is a rare disease with an incidence of 2.4 per million people in Europe [1]. Most commonly in men aged 45-65 [2]. It is a systemic vascular disease first described in 1866 by Kussmaul and Maier with symptoms of weight loss, fever, abdominal pain, and grossly visible nodular arterial aneurysms [3]. Our current understanding is that it is a multisystemic necrotizing vascular disease affecting small to medium-sized vessels. Pathogenesis is currently unknown, but there have been links to the hepatitis B virus (HBV) which is believed to arise due to secondary immune complexes [2]. There are also instances of genetic mutations, loss-of-function mutations in CECR1 (also known as ADA2), has been described in multiple sources of literature [4-6]. The American College of Rheumatology has put forth a criterion in 1990 for PAN. Weight loss, livedo reticularis, testicular tenderness, myalgias, neuropathy, diastolic blood pressure (BP) > 90 mmHg, elevated blood urea nitrogen (BUN) or creatinine, presence of HBV, arteriographic abnormality, a biopsy of a small or medium-sized artery containing polymorphonuclear cells. The presence of three out of 10 mentioned components fulfills the criteria [7]. We present a case report in which a child was found to have PAN complicated by a hypertensive crisis.

## Case presentation

A two years and four months old boy with no significant medical history, presented with an intermittent fever that started one month earlier, measured tympanic at 39 degrees Celsius and responding to antipyretics given at home. There was no diurnal variation. The fever was associated with a runny nose, a dry cough, and night sweats. The patient also complained of mild post-prandial generalized abdominal pain, for which he did not require any analgesia. There was no change in bowel habits nor was there any vomiting. Three days prior to the development of the fever, the patient had a painful swelling in the left ankle and knee, which lead to the patient limping throughout that period. The systemic review was otherwise unremarkable. Past history is unremarkable. The patient has no risk factors for acquiring an infectious disease. He initially sought medical attention at a nearby local private hospital, where he received three types of antibiotics (IV flucloxacillin, IV cefotaxime, and oral azithromycin), with no improvement. 

Upon presentation to our hospital, the patient’s blood pressure was 116/54 mmHg, the heart rate was 148 beats per minute, respiratory rate was 28 breaths per minute, and he was afebrile at 36.4 degrees Celsius. Examination showed an irritable dehydrated child with cracked lips and no lymphadenopathy. There was bilateral lower limb pitting edema up to the tibia with no skin changes or discoloration. 

Initial investigations were taken, and it revealed that the patient had anemia (hemoglobin of 9.9 g/dl), leukocytosis (19.5 x 109 cells per liter), thrombocytosis (1,036 × 109/l), high erythrocyte sedimentation rate (ESR) of 79 mm/hr and high C-reactive protein (CRP) of 65.5 mg/l. Liver function tests and renal parameters were within normal limits. No electrolyte abnormalities were seen. Infectious workup (including tuberculosis, Epstein-Barr virus, cytomegalovirus, mycoplasma, human immunodeficiency virus, hepatitis, brucella) was negative. Immunology testing was done: antineutrophil cytoplasmic antibodies (ANCA) and antinuclear antibody (ANA) were both negative. Complements (C3, C4) were within normal limits, and anti-double-stranded DNA was normal. Immunoglobulins (immunoglobulin M, immunoglobulin A and immunoglobulin E) were all normal except for a high immunoglobulin G (15.2 µg/ml). Lymphocyte markers and oxidative burst tests were all normal. Bone marrow aspirate revealed no evidence of leukemia, granuloma, or infiltration. The left foot X-ray was normal. Skeletal survey showed no lytic bone lesions. MRI of the left ankle showed a mild reactive inflammatory process. High-resolution computed tomography (HRCT) of the abdomen and the chest revealed two wedge-shaped hypodensities on the spleen, which were interpreted as splenic infarcts. Both kidneys were normal in shape and size. The patient was then discharged on oral ibuprofen with the plan to be seen in six weeks’ time for reassessment and further evaluation.

**Figure 1:**
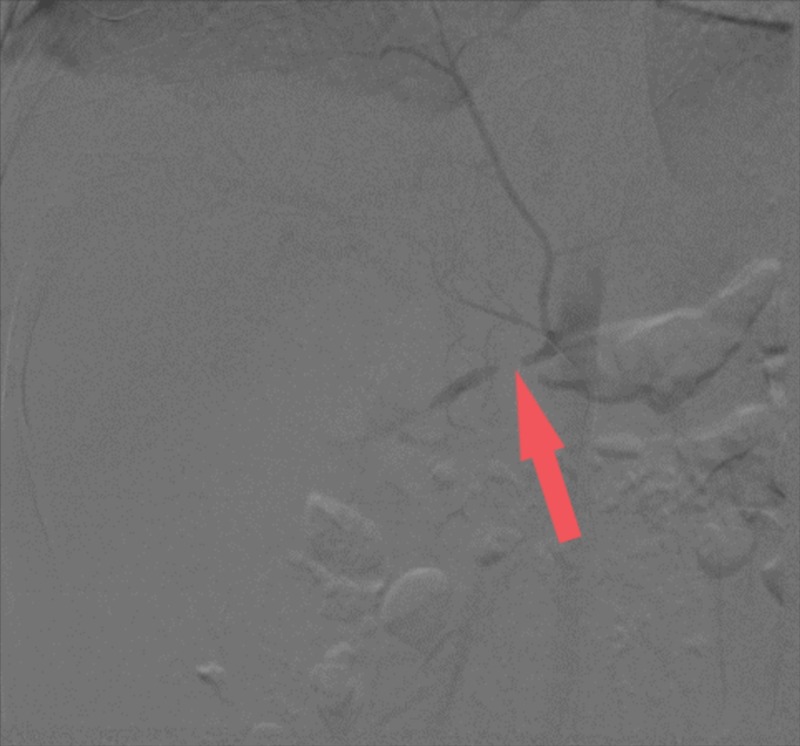
Interventional radiology angiography showing complete occlusion of the right renal artery (red arrow)

Six weeks later, the patient is readmitted. His condition was stable. He was afebrile (36.6 degrees Celsius), had no joint pain, but he had bilateral lower limb edema. Blood pressure upon this admission was 117/68 mmHg. Repeated HRCT of the chest and abdomen showed a decrease in the kidney size, post-contrast enhancement, a minimal decrease in perfusion of the right kidney, a hypodense lesion in the lower pole in addition to past findings. This was interpreted as an underlying infiltration, vasculitis, and possible granulomatous disease. Doppler ultrasound showed poor right kidney blood flow. CT angiography (Figure 1) showed that the splenic artery is completely occluded 2 cm from its origin, right renal artery occlusion 2 cm from its origin, tight stenosis of the short segment is seen at the origin of the left iliac artery, left anterior tibial artery occlusion with retrograde filling at its origin, and the distal one-third of the right posterior tibial artery showed no flow. Dimercaptosuccinic acid (DMSA) scan revealed a small hypo-functioning right kidney, normal functioning left kidney with compensatory hypertrophy. Echocardiography was done and is normal.

On the second day of admission, his blood pressure was found to be 150/98 mmHg. He was shifted to the Pediatric Intensive Care Unit (PICU), and his blood pressure was controlled initially on amlodipine (3 mg), prazosin (2 mg), clonidine (90 mcg), and hydralazine when needed. He was discharged from the PICU after stabilization. Even though the patient was kept on the three aforementioned antihypertensives, he had a relapse of high blood pressure above the 99th percentile for his height. He started to develop a gradually increasing edema, manifesting as ascites, sacral edema, scrotal edema, with upper and lower limb edema. He also gained a total of three kilograms in his weight and developed proteinuria (+4 on urinalysis, 600 mg), hypoalbuminemia (22 g/dL), and hyponatremia (126 mg). He was shifted back to the PICU, his antihypertensive medications were increased to the maximum dose, minoxidil was added and he was started on labetalol infusion 50 mL. He was given three doses of intravenous albumin (0.5 g) along with furosemide. His blood pressure was reduced to 117/71 mmHg. Repeated urinalysis showed +1 sugar, +3 protein, granular cast, with a negative culture.

He was given pulse therapy consisting of methylprednisolone 30 mg per kg per day for five days, cyclophosphamide 250 mg, infliximab 5 mg per kg infusion once, methotrexate 7.5 mg subcutaneously given weekly along with folic acid 5 mg orally. The patient was kept on oral prednisolone 2 mg per kg per day as maintenance. He underwent right renal balloon angioplasty and was successful with recanalization of the right renal artery. At this point, his edema greatly improved, blood pressure was in the range of 120/70 mmHg. The right renal doppler was performed and showed a gradual but significant improvement of the blood flow in the right renal artery.

## Discussion

Vasculitis is a systemic inflammatory process involving the blood vessels. Generally, vasculitis could be categorized as primary or secondary. Primary vasculitis can be characterized by the type and size of vessels affected, pathological changes of said vessel, and the clinical manifestations that may arise as a consequence. Common examples of primary vasculitis include Wegener's granulomatosis, microscopic polyangiitis, Churg-Strauss syndrome, and Takayasu arteritis [8]. Secondary vasculitis, on the other hand, can be caused by an underlying disease, most commonly the hepatitis B virus [2].

Polyarteritis Nodosa (PAN) is a rare necrotizing primary vasculitis that causes systemic inflammation and targets small to medium-sized vessels [9]. Symptoms may vary from person to person, but the most commonly reported symptoms upon presentation with PAN include constitutional symptoms (fever, weight loss, fatigue, weakness, and loss of appetite), joint and muscular pain, and abdominal pain [10]. 

The Diagnosis of PAN is usually confirmed with a biopsy or angiography; however, it may be suspected clinically when correlated with laboratory findings. Symptoms are nonspecific and may be applied to different types of vasculitis. These symptoms may include constitutional symptoms (fever, fatigue, weight loss, weakness, and loss of appetite), skin manifestations, such as livedo reticularis, tender erythematous nodules, purpura, and ulcers. Renal involvement may be seen as lower limb edema and hypertension. Later on, the patient may experience gastrointestinal involvement, manifested by abdominal pain, nausea, vomiting, and diarrhea [11]. Some patients may have neurological system involvement and may experience both motor and sensory impairments. Radial, ulnar and peroneal nerves are most commonly involved. Interestingly, PAN has a tendency to avoid pulmonary involvement [2].

No single pattern of laboratory findings can be established to diagnose PAN. Initial laboratory markers may show elevated acute phase reactants (ESR and CRP), normocytic normochromic anemia, and may include an abnormal renal profile and liver profile if there is an associated hepatitis infection. Urinalysis may show proteinuria. Hepatitis markers should be done to exclude secondary causes of PAN. Immunological labs should be negative for ANCA and ANA, the presence of both of which should steer the diagnosis away from PAN. Complement levels in PAN would be elevated in the case of a hepatitis infection [12]. Our patient’s laboratory findings are consistent with the literature description of PAN labs, however, there was no hepatitis infection that could be identified.

As mentioned above, the diagnosis of PAN can be confirmed by a biopsy. Angiography can be used as an alternative to a biopsy. in our case, the diagnosis was suspected after the patient developed the hypertensive crisis. CT angiography was performed, and the typical finding of a complete occlusion of the right renal artery [2].

The American College of Rheumatology (ACR) has established criteria that would help researchers and clinicians identify and suspect polyarteritis nodosa. This criteria’s main purpose is to differentiate and classify cases as PAN versus other types of vasculitis. Thus, it is mainly used for research purposes rather than clinical diagnosis. A minimum score of 3/10 is required to classify a case as PAN (Table 1). When the ACR criteria are applied to our patient, the score is 3/10, since he developed hypertension, had abnormal renal function tests, and angiography showed an occluded right renal artery [7].

**Table 1:** American College of Rheumatology 1990-year criteria for the classification of polyarteritis nodosa BP - blood pressure; BUN - blood urea nitrogen; PMN - polymorphonuclear cells

Criterion	Definition
Weight loss (24 kg)	Loss of 4 kg or more of body weight since the illness began, not due to dieting or other factors.
Livedo reticularis	Mottled reticular patterns over the skin of portions of the extremities or torso.
Testicular pain or tenderness	Pain or tenderness of the testicles, not due to infection, trauma, or other causes.
Myalgias, weakness, or leg tenderness	Diffuse myalgias (excluding shoulder and hip girdle) or weakness of muscles or tenderness of leg muscles.
Mononeuropathy or polyneuropathy	Development of mononeuropathy, multiple neuropathies, or polyneuropathy.
Diastolic BP >90 mm Hg	Development of hypertension with the diastolic BP higher than 90 mm Hg.
Elevated BUN or creatinine	Elevation of BUN >40 mg/dl or creatinine >1.5 mg/dl, not due to dehydration or obstruction.
Hepatitis B virus	Presence of hepatitis B surface antigen or antibody in the serum.
Arteriographic abnormalities	Arteriogram showing aneurysms or occlusions of the visceral arteries, not due to arteriosclerosis, fibromuscular dysplasia, or other noninflammatory causes.
Biopsy of a small or medium-sized artery containing PMN	Histologic changes showing the presence of granulocytes, or granulocytes and mononuclear leukocytes in the artery wall.

Pediatric hypertension is estimated to have a prevalence of 2-5% globally, according to Patel NH et al. Similar to adult hypertension, pediatric hypertension may manifest as a hypertensive crisis, which includes a hypertensive emergency and urgency. Hypertensive emergency is defined as having an acute elevation in systolic or diastolic blood pressure that is associated with end-organ damage, such as heart, kidneys, and the central nervous system. Hypertensive urgency shares the same definition, though it occurs without end-organ damage and may simply manifest with a headache and/or vomiting [13].

It is not unusual for PAN to present with elevated blood pressure, as O'Connell MT et al. has mentioned. They reported three cases of PAN which presented initially with a hypertensive crisis, some of which were associated with encephalopathy [14]. In contrast, our patient initially presented with a one-month history of fever, and subsequently developed a hypertensive crisis. No encephalopathy or neurological manifestations were noted. The patient’s high blood pressure was very difficult to control before establishing the diagnosis of PAN and commencing its management. Once adequate management of PAN was initiated, the patient’s blood pressure stabilized, and his condition improved significantly. The mechanism in which PAN leads to high blood pressure is believed to be mostly related to renal involvement. As the renal arteries get involved, the blood flow to the kidneys decreases with an end result of elevated blood pressure [2].

Ribi C et al. have indicated that PAN’s mainstay treatment is corticosteroids. In their study, steroids were able to maintain remission in half of their population. In those who were resistant to corticosteroids, which represented 40% of their population, azathioprine or pulse cyclophosphamide was able to achieve remission in those patients [15]. Rituximab may be used on top of the aforementioned drugs in refractory cases, a study done by Seri Y et al. has shown [16]. In our patient’s case, initial steroid therapy was unable to induce remission, thus cyclophosphamide was given, in addition to one dose of 50 mg rituximab, after which our patient’s blood pressure was easier to control. Renal angioplasty was indicated to relieve the stenosis in the renal artery.

## Conclusions

Polyarteritis nodosa is a rare but deadly disease that needs prompt diagnosis. Fortunately, enough research has been able to help physicians identify these cases and prevent serious complications from occurring. We have reported a case in which PAN was seen in a pediatric patient, complicated by a hypertensive crisis unresponsive to conventional therapy, and finally being reduced back down after identifying the underlying culprit.
